# Cartas al editor

**Published:** 2021-06-15

Bogotá, 9 de junio de 2021 

Señores

Comité Editorial

Revista Biomédica 

La ciudad

Estimados señores:

El año pasado publicamos dos artículos en *Biomédica* en los que se estudiaron los sistemas de vigilancia en salud pública en Colombia y sus departamentos usando la ley de Benford, con el fin de evaluar la calidad de los datos reportados ^1^^,^^2^. Como consecuencia de la lista clasificatoria, en algunos departamentos se suscitó una controversia por cuanto los hallazgos revelaban limitaciones en torno a la calidad objetiva de los reportes, una de las labores de salud pública fundamentales para el manejo adecuado de una epidemia. Más allá de esto, que a nuestro juicio es una crítica positiva que contribuye a definir mejores estrategias sanitarias, emergieron inquietudes sobre los factores determinantes que subyacen a estas diferencias sobre la capacidad de manejo de la pandemia en cada departamento.

Las hipótesis iniciales sugirieron que dichos factores determinantes estaban probablemente asociados con características o circunstancias propias de la búsqueda y el diagnóstico de los casos de infección por SARS- CoV-2, cuya técnica analítica se soporta en pruebas de reacción en cadena de la polimerasa con transcriptasa inversa (*Reverse Transcriptase quantitative* PCR, RT-qPCR). Epidemiológicamente, la detección de casos se fundamenta en la búsqueda activa de contactos de los casos conocidos (fruto del llamado “cerco epidemiológico”), en el diagnóstico fortuito cuando la prueba se hace por alguna necesidad propia de cada persona (casos asintomáticos), y el diagnóstico clínico cuando se presentan casos que ameritan atención hospitalaria (casos sintomáticos), principalmente. Es decir, corresponde a actividades de “epidemiología de campo”.

La otra consideración importante es la disponibilidad de laboratorios para realizar la RT-qPCR, dado que no es una prueba que convencionalmente esté disponible en los laboratorios clínicos de Colombia. Por ello, a partir de lo dispuesto por la normatividad colombiana, en especial el Artículo 16 del Decreto 2323 de 2006, en los laboratorios departamentales de salud pública recae la responsabilidad de un diagnóstico oportuno como respuesta durante las crisis sanitarias. En ese sentido, tener disponible un laboratorio con capacidad diagnóstica en el territorio resulta fundamental, pues disminuye los tiempos de respuesta. Sin embargo, a efectos de la medición del desempeño del sistema sanitario público, no es lo mismo tener a cargo del diagnóstico a un laboratorio clínico (privado o público), una institución académica o de investigación, o un laboratorio departamental de salud pública. Este último implica una serie de esfuerzos y gestiones que bien pueden informar sobre la calidad de la respuesta gubernamental, tanto en la preparación como durante la pandemia.

En este contexto, se solicitó mediante un derecho de petición dirigido al Instituto Nacional de Salud, el listado por departamento de los laboratorios con capacidad de realizar las mencionadas pruebas y la fecha de inicio de los diagnósticos. Una vez obtenida la información, se rehízo el análisis objetivo de la calidad del reporte de casos de COVID-19 considerando como variable dependiente el cumplimiento o no de la ley de Benford y como determinantes, la presencia de cualquier laboratorio con capacidad diagnóstica, el laboratorio gubernamental con capacidad diagnóstica y los tiempos entre el primer caso en el departamento y el inicio de actividades de este tipo de laboratorios. Los resultados sugirieron que los datos provenientes de los territorios donde el laboratorio gubernamental tenía la capacidad de realizar la prueba de RT- qPCR mostraban una mejor calidad, lo que sugiere un mejor desempeño de los procesos de vigilancia en salud pública (cuadro 1).

**Cuadro 1:** Asociación entre la buena calidad de los datos de la vigilancia en salud pública y la presencia de un laboratorio departamental de salud pública con capacidad para realizar pruebas de RT-qPCR en Colombia (n=33)

Variable	OR^a^	p	IC_95%_
Laboratorio estatal con capacidad de RT-qPCR*	9,63	0,0183	1,36 - 119,92
Sensibilidad (%)	77,78		
Especificidad (%)	75,00		
Clasificación correcta (%)	75,76		

^*^Odds ratio obtenido con regresión logística exacta

^*^Datos oficiales del Instituto Nacional de Salud (corte a septiembre 1° de 2020)

En Colombia, estos resultados son llamativos dado que la organización del sistema de salud prioriza las acciones individuales de atención sobre las actividades de salud pública, a la vez que las aseguradoras públicas y privadas son ampliamente criticadas por su deficiente desempeño ^3^^,^^4^.

Lo anterior refuerza la hipótesis de que un adecuado desempeño cuantitativo de la vigilancia en salud pública debe estar sustentado en la optimización de los procesos públicos de gestión e inversión, para así garantizar la prontitud en la instalación y el funcionamiento óptimo de los laboratorios de salud pública departamentales. La evidencia en este sentido es contundente en la medida en que, si la proporción de casos detectados se incrementa en 10% mediante un adecuado rastreo de contactos, la mortalidad por COVID-19 se reduciría entre 0,8 y 3,4% ^5^. Que estos resultados sirvan para definir las inversiones futuras en salud pública en los territorios, porque la crisis sanitaria actual no será la última que enfrentemos. Más vale prepararse desde ahora para las siguientes epidemias y pandemias.

Alvaro Javier Idrovo

Departamento de Salud Pública, Escuela de Medicina, Universidad Industrial de Santander, Colombia

José Moreno-Montoya

Subdirección de Estudios Clínicos, Fundación Santa Fe de Bogotá, Colombia
